# School-Based Online Surveillance of Youth: Systematic Search and Content Analysis of Surveillance Company Websites

**DOI:** 10.2196/71998

**Published:** 2025-07-08

**Authors:** Alison O'Daffer, Wendy Liu, Cinnamon S Bloss

**Affiliations:** 1 Center for Empathy and Technology, Sanford Institute for Empathy and Compassion, University of California San Diego, 9500 Gilman Drive MC 0811, La Jolla, CA, 92037-0811, United States, 1 858-534-9595, 1 858-534-9595; 2 Joint Doctoral Program of Clinical Psychology, San Diego State University/University of California San Diego, San Diego, CA, United States; 3 Rady School of Management, University of California San Diego, La Jolla, CA, United States; 4 Herbert Wertheim School of Public Health and Human Longevity Science, University of California San Diego, La Jolla, CA, United States

**Keywords:** schools, secondary schools, adolescents, youth, mental health, privacy, surveillance, monitoring

## Abstract

**Background:**

School-based online surveillance of students has been widely adopted by middle and high school administrators over the past decade. Little is known about the technology companies that provide these services or the benefits and harms of the technology for students. Understanding what information online surveillance companies monitor and collect about students, how they do it, and if and how they facilitate appropriate intervention fills a crucial gap for parents, youth, researchers, and policy makers.

**Objective:**

The two goals of this study were to (1) comprehensively identify school-based online surveillance companies currently in operation, and (2) collate and analyze company-described surveillance services, monitoring processes, and features provided.

**Methods:**

We systematically searched GovSpend and EdSurge’s Education Technology (EdTech) Index to identify school-based online surveillance companies offering social media monitoring, student communications monitoring, or online monitoring. We extracted publicly available information from company websites and conducted a systematic content analysis of the websites identified. Two coders independently evaluated all company websites and discussed the findings to reach 100% consensus regarding website data labeling.

**Results:**

Our systematic search identified 14 school-based online surveillance companies. Content analysis revealed that most of these companies facilitate school administrators’ access to students’ digital behavior, well beyond monitoring during school hours and on school-provided devices. Specifically, almost all companies reported conducting monitoring of students at school, but 86% (12/14) of companies reported also conducting monitoring 24/7 outside of school and 7% (1/14) reported conducting monitoring outside of school at school administrator-specified locations. Most online surveillance companies reported using artificial intelligence to conduct automated flagging of student activity (10/14, 71%), and less than half of the companies (6/14, 43%) reported having a secondary human review team. Further, 14% (2/14) of companies reported providing crisis responses via company staff, including contacting law enforcement at their discretion.

**Conclusions:**

This study is the first detailed assessment of the school-based online surveillance industry and reveals that student monitoring technology can be characterized as heavy-handed. Findings suggest that students who only have school-provided devices are more heavily surveilled and that historically marginalized students may be at a higher risk of being flagged due to algorithmic bias. The dearth of research on efficacy and the notable lack of transparency about how surveillance services work indicate that increased oversight by policy makers of this industry may be warranted. Dissemination of our findings can improve parent, educator, student, and researcher awareness of school-based online monitoring services.

## Introduction

### Background

School-based online surveillance of students has been adopted widely by middle and high school administrators (eg, principals, district officials, or information technology directors) over the past decade [1,2]. Purportedly designed to protect students, many school districts have used the technology with the goal of preventing youth suicide and school violence, including gun violence and cyberbullying [3]. With rising rates of youth suicide [4,5], considerable concern about gun violence in schools [6], and ongoing reports of cyberbullying [7], schools are key stakeholders in keeping adolescents safe and healthy into adulthood [8]. There are no up-to-date, publicly available data on the prevalence of school-based online surveillance in the United States, but school purchases of online surveillance services have increased annually since 2013 [1], and 9 of 10 secondary school teachers surveyed report that their schools use student online surveillance technology [9]. Research suggests that most parents, teachers, and school administrators support the use of online surveillance to detect risks of students harming themselves or others, but student attitudes are mixed [10,11].

School-based online surveillance companies are companies that sell one or more of the following services to schools or districts: social media monitoring, student communications monitoring (eg, email or documents), and online (ie, web browsing) monitoring [12]. School-based online surveillance services are a subset of the educational technology (EdTech) surveillance industry. School-based online surveillance is distinct from web filtering, which is mandated by the Children’s Internet Protection Act [13] and blocks students from viewing certain content online. School-based online surveillance is more invasive than web filtering, in that it scans students’ online activity for specific words or phrases determined to be concerning, traces flagged content to specific students’ identities, and shares that information with school staff [12].

While online surveillance of students was once limited to public social media post collation through geofencing [14-16], recent advances in artificial intelligence (AI) provide more sophisticated and intrusive methods for surveilling individuals’ online activity [17]. Under the Family Educational Rights and Privacy Act, schools can legally provide identifiable student information to contractors to perform school personnel functions [18]. Large language models can analyze a large amount of student online activity (ie, emails, web searches, direct messages, or private social media browsing and posting) if school administrators give that level of access to student data to online surveillance companies. The use of large language models for reviewing sensitive student data presents risks of AI bias for marginalized students [19]. In the school-based surveillance space, potential adverse effects of automated surveillance via AI include higher false positive rates for Black students, outing of LGBTQ+ students, or excessive discipline of students with disabilities [12].

Most school-based online surveillance services follow a similar process. First, school or district administrators purchase school-based online surveillance and give a surveillance company access to ongoing student online activities. The surveillance company then conducts automated monitoring of what students are doing online. If something a student did online is deemed problematic by the company’s automated review mechanism, the technology would then generate an alert and push it to school administrators with the name of the student and the content that was flagged. Within this general process, different companies have different nuanced approaches to how they get access to student data, how much data they can access, what automated monitoring technology they use to generate alerts, and how alerts are handled.

Despite the ubiquity of use of such technology at schools, there is little systematic knowledge about the scope and practices of the school-based online surveillance industry [12], compounding the lack of empirical evidence of the efficacy of this technology and the societal effects of this industry. It is unclear how many online surveillance companies are currently in operation, what services each company offers, and if and how AI is being leveraged by these companies. Understanding what information online surveillance companies claim to monitor and collect about students, how they claim to do it, and if and how they facilitate or claim to facilitate appropriate intervention fills a crucial knowledge gap for parents, youth, researchers, and policy makers, and may inform decision-making related to this important industry.

### Aims

We conducted a systematic search for school-based online surveillance companies and a content analysis of the websites identified. Our research questions were as follows: (1) what are the school-based online surveillance companies currently in operation in the US? (2) What surveillance services, monitoring processes, and features do school-based online surveillance companies report offering?

## Methods

### Systematic Search of Online Surveillance Companies

We sought to identify school-based online surveillance companies and conduct a content analysis of their websites. Entities were included in our analysis if they (1) claimed to monitor students’ social media, private electronic communications, or online browsing activity; (2) claimed to generate and save alerts tied to individual students’ identities; (3) had informational materials available in English; (4) specifically designed or marketed their services to schools (even if they marketed to multiple industries); and (5) were in operation at the time of the systematic search.

Identification of online surveillance companies and website content analysis was conducted from February 2024 to June 2024. Our systematic search process to identify companies was threefold. First, we searched GovSpend [20], an extensive database of US public government procurement records, to identify online surveillance companies with which school districts across the country have contracts. Public schools and districts in the United States are typically obligated to make contract information publicly available when paying nonemployees for contract work [21]. GovSpend is a commercially available data aggregation service for these public records, capturing state, local, and education spending in the United States. GovSpend reports collecting spending, purchase order, and contracts data directly from thousands of US public agency partners every quarter. We searched K-12 school contract data in the GovSpend database for online surveillance companies, using general keywords “computer monitoring,” “social media monitoring,” “EdTech,” “risk detection,” and specific names of companies we know of from news stories and websites of school-based online surveillance companies.

Second, we used EdSurge’s EdTech Index tool [22], an extensive index of EdTech products and services, to search for online surveillance companies. EdSurge’s EdTech Index uses categorical labels to sort EdTech services, so we screened all services labeled “wellness and mental health” or “learning technology management” for “K-12 education.”

To ensure we captured all school-based online surveillance companies, we augmented these search strategies by reviewing news articles mentioning school-based online surveillance through Google News over the past 10 years. Our team was concurrently conducting qualitative work with school administrators, adolescents, and parents of adolescents during the time of this analysis, so we also cross-referenced any company mentioned in those interviews with our list from GovSpend and EdSurge’s EdTech Index.

The resulting initial set of companies was then subjected to further screening, with exclusions made if (1) the company did not conduct student communications monitoring, social media monitoring, or online monitoring; (2) the company’s monitoring data was not linked to student identities; (3) the company did not design or market their services to schools (even if they marketed to other industries); or (4) the company was no longer in operation. No additional companies were identified through our review of news articles or concurrent qualitative interviews, but 1 company, Sergeant Laboratories, was identified as providing school-based online surveillance by a school administrator after being screened and excluded previously. Sergeant Laboratories was lacking information on their website, which likely contributed to the initial exclusion.

Once we evaluated each company using these criteria, we arrived at the final set of companies for analysis. After the final companies were identified, we extracted information from LinkedIn about each company, including the location of the headquarters, year founded, approximate number of employees, and target customers.

### Content Analysis

For each online surveillance company that met the final inclusion criteria, we extracted publicly available information from the company’s website and saved all website content in our files. All website information was extracted between February and June 2024. We developed a codebook specifically for this project (Multimedia Appendix 1). The codebook was created via an iterative, deductive process. First, based on existing literature, we developed research questions and codes on the 3 topic areas of interest a priori (types of services, monitoring processes, and features). While conducting data extraction, additional codes were added as needed (eg, if an online surveillance company claimed to offer a feature we had not heard of or included in our original codebook, we added that code to our codebook to capture the information). In our codebook, 40 codes were chosen by the authors within the 3 topic areas related to our research questions: types of services, monitoring processes, and features. Types of services codes included whether each company conducted student communications monitoring, social media monitoring, or online activity monitoring. Monitoring process codes included access mechanisms, monitoring mechanisms, types of software screened, when and where monitoring is conducted, and types of devices and accounts monitored. Codes about features offered included content, alert generation, alert response, and student tracking features.

Two coders, an author and a research associate (AO and Natalie Gonzalez, BA), independently reviewed data for all 14 company websites and independently labeled each variable from our codebook. We met to discuss findings, review incongruities, and reach 100% consensus. Consensus conversations took place in May 2025.

## Results

### Systematic Search of Online Surveillance Companies

**Figure 1.**
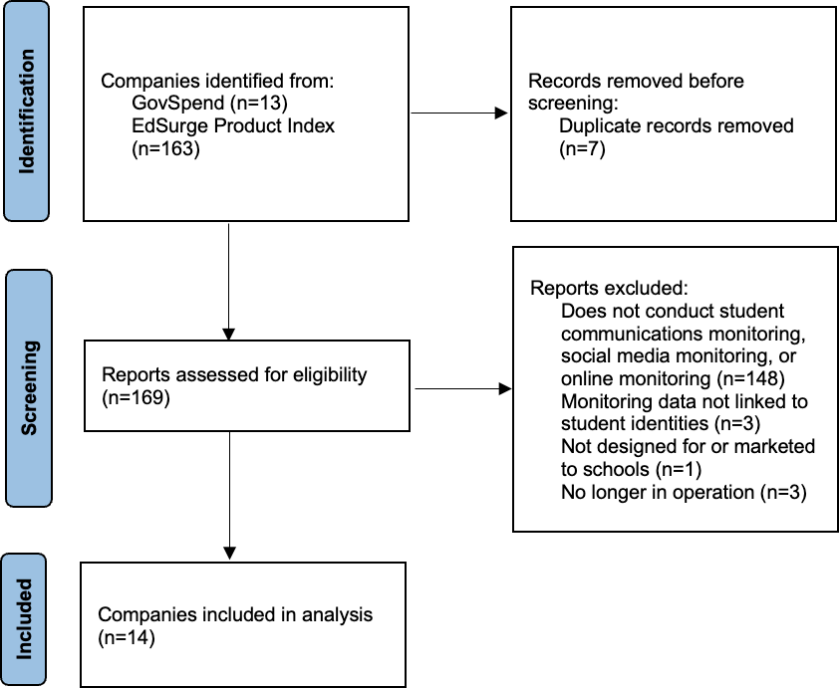
PRISMA flow diagram [37] for systematic search of online surveillance companies. PRISMA: Preferred Reporting Items for Systematic Reviews and Meta-Analyses.

The keyword search in GovSpend and EdSurge yielded 169 companies that could potentially offer school-based surveillance services. Of these, 148 companies were then screened out because they did not claim to monitor students’ social media, private electronic communications, or online browsing activity. Four companies were excluded because they did not claim to generate alerts tied to students’ individual identities. Three companies were excluded because they were no longer in operation. No companies were excluded due to not having materials in English, likely because we searched English-language databases. After all screening criteria were applied, we identified 14 school-based online surveillance companies [23-36]. The PRISMA flow diagram [37] (Figure 1) provides more detailed information about our search and screening process.

The 14 online surveillance companies identified and included in the content analysis are Ativion [23], Bark [24], Blocksi [25], Deledao [26], Gaggle [27], GoGuardian [28], Lightspeed Systems [29], Linewize by Qoria [30], ManagedMethods [38], Navigate360 [32], Netsweeper [33], Safer Schools Together [34], Securly [35], and Sergeant Laboratories [36]. Information about these companies can be found in Multimedia Appendix 2.

One company, Sergeant Laboratories, conveyed that they monitor online activity, but had extremely limited information about their services available on their website [36]. Despite multiple contact attempts, they ultimately declined to provide additional information, which explains the gaps in information in the Results and Tables for Sergeant Laboratories.

### Content Analysis

#### Services Offered and Pricing

Our content analysis revealed that 12/14 (86%) companies offered student communications monitoring [23-32,35], 7/14 (50%) companies offered social media monitoring [26,29,30,32-35], 11/14 (79%) companies offered online monitoring [23-31,33,35], and 1/14 (7%) companies did not provide information about the specific digital activity they monitor [36]. Table 1 details surveillance services offered by the 14 school-based online surveillance companies included in our analysis.

**Table 1.** Surveillance services offered by school-based online surveillance companies (N=14).

Company	Student communications monitoring^a^ (n=12)	Social media monitoring^b^ (n=7)	Online monitoring^c^ (n=11)	Unclear or unspecified (n=1)
Ativion [23]	✓		✓	
Bark [24]	✓		✓	
Blocksi [25]	✓		✓	
Deledao [26]	✓	✓	✓	
Gaggle [27]	✓		✓	
GoGuardian [28]	✓		✓	
Lightspeed Systems [29]	✓	✓	✓	
Linewize by Qoria [30]	✓	✓	✓	
ManagedMethods [31]	✓		✓	
Navigate360 [32]	✓	✓		
Netsweeper [33]	✓	✓	✓	
Safer Schools Together [34]		✓		
Securly [35]	✓	✓	✓	
Sergeant Laboratories [36]				✓

a Student communications monitoring: technology that “scans private student electronic communications, such as emails and documents written on school accounts and software applications, for words and phrases deemed by the technology provider [or] school to be problematic and shares concerning communications with the provider and/or school” [12].

b Social media monitoring: technology that “scans students’ public social media accounts for words and phrases that are designated by the school [or] the product provider to be problematic, even when they are off campus” [12].

c Online monitoring: technology that “monitors what students search for online and what websites they visit, and flags concerning activities for the technology provider [or] school” [12].

Pricing information was not publicly available for 93% (13/14) of online surveillance companies [23,25-36]; the exception was Bark, which provides its basic services to schools for free and offers a more in-depth, paid, parent platform, and paid add-ons for schools [24]. A total of 43% (6/14) of companies had information on their website about how to apply for governmental grants to pay for online surveillance [24,27-30,32].

#### Monitoring Processes

Online surveillance companies reported obtaining access to student data through a variety of mechanisms. Table 2 provides detailed information about access mechanisms for each of the 14 school-based online surveillance companies. A total of 4/14 (29%) companies reported accessing student data through software installed on school-issued devices [23,28-30], and 3/14 (21%) companies used browser plug-ins or extensions (software installed on web browsers) [23,24,30]. Further, 7/14 (50%) companies reported accessing student information through school-provided account access [24,25,27,28,30,31,35]. Additionally, 3/14 (21%) companies reported accessing information through application programming interface integrations (ie, software interfaces that connect computers or computer software without needing to install anything on a specific device or device’s browser) [29,31,32]. Furthermore, 3/14 (21%) companies reported accessing student information through publicly available online content [32,34,35]. Moreover, 3/14 (21%) companies either provided unclear information about access or did not specify how they access student data [26,33,36]. Besides, 8/14 (57%) companies reported using more than one of these methods to access student online activity data [23,24,28-32,35].

**Table 2.** Monitoring mechanisms used by school-based online surveillance companies (N=14).

Company	How does the company access student data?						How does the company screen student data?		
							Automated methods		Nonautomated methods
	Browser plug-in^a^ (n=3)	Device software or app (n=4)	School-provided account access (n=7)	API integr-ation^b^ (n=3)	Public online activity (n=3)	Unclear or not specified (n=3)	Artificial intelligence (n=10)	Unspecified automated mechanism (n=4)	Human review team (n=6)
Ativion [23]	✓	✓					✓		
Bark [24]	✓		✓				✓		✓
Blocksi [25]			✓				✓		✓
Deledao [26]						✓	✓		
Gaggle [27]			✓				✓		
GoGuardian [28]		✓	✓					✓	✓
Lightspeed Systems [29]		✓		✓			✓		✓
Linewize by Qoria [30]	✓	✓	✓					✓	✓
ManagedMethods [31]			✓	✓			✓		
Navigate360 [32]			✓	✓	✓		✓		
Netsweeper [33]						✓	✓		✓
Safer Schools Together [34]					✓			✓	
Securly [35]				✓	✓		✓		
Sergeant Laboratories [36]						✓		✓	

a Browser plug-in: software installed onto a web browser.

b API integration: Application programming interface integration refers to software interfaces that connect computers or computer software without needing to install anything on a specific device or device’s browser.

Many companies reported surveilling specific software. Table 3 details specific software and applications that the 14 school-based online surveillance companies reported monitoring. Specifically, 10/14 (79%) online surveillance companies said they monitored student activity on Google Workspace [24-33,35], 10/14 (71%) said they monitored student activity on Microsoft 365 [24,25,27-33,35], and 10/14 (71%) said they monitored student web browser activity [23-30,33,35]. Further, 3/14 (21%) online surveillance companies reported monitoring student activity on Canvas, a web-based learning management system [27,33,35]. One company, Netsweeper, reported monitoring student activity on smartphone apps [29]. Furthermore, 3/14 (21%) online surveillance companies reported monitoring public social media activity [32,34,35], while 5/14 (36%) companies reported monitoring all social media activity [26,29,30,33,35]. One company, Sergeant Laboratories, did not provide any information about the software it surveils [36].

**Table 3.** Software screened by school-based online surveillance companies (N=14).

Company	Student communications				Social media		Online activity
	Google Workspace (n=11)	Microsoft 365 (n=10)	Canvas (n=3)	Public social media posts (n=3)	All social media activity (n=5)	Web browsers (n=10)	Smartphone apps (n=1)
Ativion [23]						✓	
Bark [24]	✓	✓				✓	
Blocksi [25]	✓	✓				✓	
Deledao [26]	✓				✓	✓	
Gaggle [27]	✓	✓	✓			✓	
GoGuardian [28]	✓	✓				✓	
Lightspeed Systems [29]	✓	✓			✓	✓	
Linewize by Qoria [30]	✓	✓			✓	✓	
ManagedMethods [31]	✓	✓					
Navigate360 [32]	✓	✓		✓			
Netsweeper [33]	✓	✓	✓		✓	✓	✓
Safer Schools Together [34]				✓			
Securly [35]	✓	✓	✓	✓	✓	✓	
Sergeant Laboratories^a^ [36]							

a Sergeant Laboratories had incomplete information on their website. We reached out to the company for more information, and they ultimately declined to comment.

A total of 93% (13/14) of companies reported conducting monitoring of students at school [23-35]. Locations, devices, and accounts monitored by online surveillance companies are detailed in Table 4. Further, 86% (12/14) of companies reported conducting monitoring 24/7 at any location outside of school [23-33,35] and 7% (1/14) reported conducting monitoring outside of school only in administrator-specified locations [34]. Additionally, 93% (13/14) of online surveillance companies indicated that they monitored student activity on school-issued devices [23-33,35,36], 36% (5/14) reported that they monitored at least some student activity on student-owned computers and tablets [25,27,32,33,35], and 36% (5/14) reported that they monitored at least some student activity on student-owned smartphones [25,27,32,33,35]. One online surveillance company, Safer Schools Together, only reported monitoring public social media activity of students, and therefore did not monitor any specific devices [34]. Moreover, 86% (12/14) of companies reported monitoring school-provided accounts [23-33,35], and 43% (6/14) reported monitoring at least some activity on personal accounts [26,30,32-35].

**Table 4.** Locations, devices, and accounts monitored by school-based online surveillance companies (N=14).

Company	Where is monitoring conducted?			What devices are monitored?			What accounts are monitored?	
	At school (n=13)	Any location outside of school (n=12)	Specified locations outside of school (n=1)	School-issued devices (n=13)	Student-owned computers or tablets (n=5)	Student-owned cell phones (n=5)	School- provided accounts (n=12)	Personal accounts (n=6)
Ativion [23]	✓	✓		✓			✓	
Bark [24]	✓	✓		✓			✓	
Blocksi [25]	✓	✓		✓	✓	✓	✓	
Deledao [26]	✓	✓		✓			✓	✓
Gaggle [27]	✓	✓		✓	✓	✓	✓	
GoGuardian [28]	✓	✓		✓			✓	
Lightspeed Systems [29]	✓	✓		✓			✓	
Linewize by Qoria [30]	✓	✓		✓			✓	✓
ManagedMethods [31]	✓	✓		✓			✓	
Navigate360 [32]	✓	✓		✓	✓	✓	✓	✓
Netsweeper [33]	✓	✓		✓	✓	✓	✓	✓
Safer Schools Together [34]	✓		✓					✓
Securly [35]	✓	✓		✓	✓	✓	✓	✓
Sergeant Laboratories^a^ [36]				✓				

a Sergeant Laboratories had incomplete information on their website. We reached out to the company for more information, and they ultimately declined to comment.

All 14 online surveillance companies reported using some sort of automated technology, particularly AI, to analyze the data from monitoring and to generate alerts (Table 2). Furthermore, 71% (10/14) of online surveillance companies reported using AI to conduct automated flagging of student activity [23-27,29,31-33,35]. Additionally, 29% (4/14) of companies did not clarify how they generate automated alerts [28,30,34,36]. Some companies mentioned more specific applications of AI as part of monitoring: 14% (2/14) of companies reported using machine learning (an application of AI) [23,27] and 14% (2/14) reported using natural language processing (a type of machine learning) [24,35]. Some online surveillance companies also reported using AI for sentiment analysis (1/14, 7%) [34], keyword analysis (2/14, 17%) [23,35], and image analysis (1/14, 7%) [35]. In addition to automated monitoring technology, 43% (6/14) of online surveillance companies reported having a secondary human review team to review automated alerts [24,25,28-30,33].

#### Features Offered

Specific features offered by online surveillance companies are detailed in Multimedia Appendix 3. Further, 86% (12/14) of companies reported providing an alert management dashboard to view and track responses to alerts [23-33,35]. Furthermore, 71% (10/14) of companies reported providing after-hours alerts for prespecified school administrators [23-25,27-31,33,35], and 71% (10/14) of companies reported having company staff on call after hours to support school personnel who respond to student alerts [23-25,27-29,33-35]. One (of 14) online surveillance company, Securly, reported detecting harassing content as students type and “nudging” students to think twice before posting or sending [35]. Additionally, 29% (4/14) of companies reported generating individual student “wellness” or “risk” scores and providing data visualization tools for school administrators to view scores (eg, at individual, classroom, grade, and school levels) [23,24,30,35]. Moreover, 43% (6/14) of companies reported offering a complementary parent platform for parents to view their child’s individual information and alerts [24-26,29,30,35]. Two (14%) companies, Gaggle and Netsweeper, reported contacting law enforcement “as needed” after hours to respond to student alerts [27,33]. One online surveillance company, Gaggle, reported that they offer virtual crisis response by company staff [27]. One online surveillance company, Navigate360, reported offering online psychoeducation modules for students identified via monitoring as at mental health risk [32].

## Discussion

### Principal Findings

This study provides the first detailed assessment of the current school-based online surveillance industry. Our systematic search and content analysis found that all 14 companies identified offer extensive monitoring of school-issued devices and accounts. School-based monitoring of personal devices and accounts is offered by over half of the companies, but these services seem to have gaps in data access. Most companies reported using AI to process student data, and algorithmic transparency is lacking. Less than half of school-based online surveillance companies have human staff review AI-generated alerts before alerts are sent to school staff.

As this paper is the first to outline the scope and offerings of this industry in detail, we suspect that there is low stakeholder and public awareness about this technology. Dissemination of our findings can improve public awareness of school-based online monitoring services. This study also suggests that students who only have school-issued devices (eg, low-income students) are likely being surveilled more heavily than students who have access to personal devices. Established research suggests that large language models demonstrate notable biases against non-White groups [19], so reliance on AI for monitoring without algorithmic transparency presents risks of discrimination or adverse outcomes for historically marginalized students. Companies without human review teams (Ativion [23], Deledao [26], Gaggle [27], ManagedMethods [31], Navigate360 [32], Safer Schools Together [34], Securly [35], and Sergeant Laboratories [36]) may be at a higher risk of generating false positive alerts that lead to unwarranted disciplinary procedures for students [39]. To reduce these risks, surveillance companies should use methods to identify AI bias (eg, data audits, fairness metrics, or adversarial testing) and mitigate it (eg, algorithmic fairness techniques, training models on diverse data, or continuous human oversight) [40-42], while providing transparent information to consumers.

There is a notable lack of transparency about how surveillance companies work and a dearth of research on whether these school-based online surveillance products improve student outcomes and prevent harms [12,43,44]. Research on other types of school-based online surveillance (eg, security guards, cameras, or metal detectors) suggests that school surveillance is ineffective in preventing violence [45,46] and has negative effects on youth mental health [47-52], adding to concern about broad implementation of online surveillance technology in schools. The dearth of empirical research on online surveillance services is especially concerning given the serious topics facing students that online surveillance companies claim to prevent (eg, suicidality, self-harm, cyberbullying, and gun violence). Without transparency and accountability, this industry is susceptible to unproductive competition and even deceptive practices. Federal education grants are often used to pay for school-based online surveillance services [53], but at present, the lack of research or reliable metrics makes it difficult to determine whether this technology is an appropriate use of federal funds. Further, any gains in school safety from this technology should be assessed in balance to the potential loss in privacy and cybersecurity risks.

Excessive surveillance may lead to a loss of students’ intimate privacy [43] which can impede their learning, social development, and mental health. Sharing of student data with outside companies also exposes such data to wider cybersecurity attacks. The sensitivity of the topic area, an opaque market, a lack of empirical assessment of effectiveness, and the growing prowess of data extraction and analysis are concerning. These factors, taken together with trade-offs of decreased privacy and increased cybersecurity risks, suggest that the school-based online surveillance industry may require more oversight from regulators, greater monitoring from the public, and more thorough risk assessment. Policy options to mitigate risks may include federal regulations on AI bias, transparency requirements for surveillance companies, and opt-in mechanisms for student monitoring outside of school hours.

These companies’ extensive access to students’ online data activity 24/7 also raises questions regarding the role of the school versus the role of parents in monitoring and controlling minors’ technology use. It is unclear to what extent students, parents, and guardians understand or even are aware of schools’ use of surveillance technology. Our team had challenges accessing detailed, clear information about these companies, suggesting it is currently difficult for students and parents to find information to make informed decisions. More attention should be given to the issue of how to communicate the relevant information and craft opt-in and opt-out options to parents for school-based digital surveillance.

While this research analyzed the scope and nature of surveillance services currently provided to schools, an important question that remains to be further examined is why are we observing this particular structure of service offerings. In particular, it could be the result of the intentions of both the suppliers and the customers (schools). For example, 1 possibility is that the surveillance companies developed a comprehensive menu of services in terms of surveillance method, scope, and frequency, and schools choose what they deem appropriate and affordable from the menu. If this were the case, then what we are observing largely reflects the schools’ preferences for what to monitor and how to use the information. In contrast, another possibility is that the surveillance technology companies decide on what services to offer based on their own knowledge of what monitoring is effective, as well as their financial incentives, such as the profitability of each type of service and the legal and reputational risks involved. They then actively persuade schools to adopt certain types of services. The eventual market outcome is likely a result of both dynamics. Future research can investigate to what extent schools versus surveillance service providers have greater influence in the service selection decisions.

### Limitations

We have identified a thorough list of companies through GovSpend and EdSurge’s EdTech Index, reviewing news articles mentioning school-based online surveillance, and cross-referencing with qualitative data from other ongoing work in our research program. However, it is possible that our search strategy missed one or more companies due to gaps in databases or news coverage. Similarly, we limited our analysis to companies that market to schools, so we may be underestimating the scope and scale of the school-based online surveillance industry. We limited this analysis to companies that market to schools because school settings are distinct from other surveillance use cases (eg, law enforcement).

With the rapidly changing landscape of technology companies, the content of these company websites may have changed in the time since we conducted data extraction. This analysis does not include 3 online surveillance companies (GeoListening, GeoFeedia, and SnapTrends) that are no longer in operation.

The exclusive reliance on publicly available website content for our content analysis may lead to incomplete or skewed representations of what school-based online surveillance services actually do in practice. Websites are often marketing tools, and companies may not disclose all relevant information or may present their services in a favorable light. Publicly available information on websites may not reflect internal policies or contract terms. However, we chose to rely solely on company website data because that is what school administrators purchasing these services are exposed to, and to ensure reproducibility of this study and lay the foundation for future work evaluating claims made by school-based online surveillance companies. Future research to evaluate these claims may include qualitative interviews with stakeholders, analysis of school contracts, or testing surveillance systems in practice.

### Conclusions

School-based online surveillance companies all claim to closely monitor student activity on school-provided devices and accounts, and many also claim to monitor personal devices and accounts. Despite a lack of evidence for the effectiveness of these products, these companies nonetheless have extensive reach into the digital lives of students and their families. Further, federal funds are often used to pay for these services that monitor student activity 24/7 and use opaque algorithms to process student data. This paper can serve as an informative tool for parents, youth, researchers, and regulators curious about the scope and depth of school-based online surveillance services who are seeking to make informed decisions about this technology.

## Supplementary material

10.2196/71998 Multimedia Appendix 1 Codebook.

10.2196/71998 Multimedia Appendix 2 LinkedIn information about school-based online surveillance companies.

10.2196/71998 Multimedia Appendix 3 Features offered by school-based online surveillance companies (N=14).

## Abbreviations

AI: artificial intelligence
EdTech: Educational Technology
