# Supplementary material for: School-Based Online Surveillance of Youth: Systematic Search and Content Analysis of Surveillance Company Websites
Source: J Med Internet Res. 2025 Jul 8;27:e71998. doi: 10.2196/71998 (PMC12262101; doi:10.2196/71998)
Supplement: Multimedia Appendix 1 [file jmir-v27-e71998-s001.docx]

| **Category** | **Topic** | **Codes** |
| --- | --- | --- |
| Services offered | Surveillance services | Student communications monitoring  Social media monitoring  Online monitoring |
| Monitoring processes | Access mechanism(s) | Browser plug-in |
|  |  | Device software or app |
|  |  | Software integration |
|  |  | School-provided account access |
|  |  | Public online activity |
|  |  | Unclear/not specified |
|  | Monitoring mechanism(s) | Artificial intelligence   - - - - Machine learning       - Natural language processing       - Sentiment analysis       - Keyword analysis       - Image analysis   Unspecified automated mechanism |
|  |  | Human review team |
|  | Programs screened | Productivity software   - Google Workspace - Microsoft 365 - Canvas |
|  |  | Social media   - Public social media posts - All social media activity |
|  |  | General internet use   - Web searches - Smartphone apps |
|  | When monitoring is conducted | At school |
|  |  | Specified locations outside of school |
|  |  | Any location outside of school |
|  | Devices monitored | School-issued devices |
|  |  | Student-owned computers and tablets |
|  |  | Student-owned cell phones |
|  | Accounts monitored | School-provided accounts |
|  |  | Personal accounts |
| Features offered | Content review features | Anti-harassing content nudge |
|  | Alert features | After hours alerts for school administrators |
|  |  | Company staff on call after hours |
|  |  | Alerts about active planning of suicide |
|  | Tracking features | Alert management dashboard |
|  |  | Student wellness/risk scores |
|  |  | Parent platform |
