# Supplementary material for: School-Based Online Surveillance of Youth: Systematic Search and Content Analysis of Surveillance Company Websites
Source: J Med Internet Res. 2025 Jul 8;27:e71998. doi: 10.2196/71998 (PMC12262101; doi:10.2196/71998)
Supplement: Multimedia Appendix 2 [file jmir-v27-e71998-s002.docx]

| **Company** | **Headquarters** | **Year Founded** | **# of Employees on LinkedIn** | **Products Marketed to:** | | |
| --- | --- | --- | --- | --- | --- | --- |
|  |  |  |  | **Schools** | **Parents** | **Businesses** |
| **Ativion** | New York, New York | 2001 | 51-200 | ✅ | ✅ | ✅ |
| **Bark** | Kaysville, Utah | 2015 | 51-200 | ✅ | ✅ |  |
| **Blocksi** | Palo Alto, California | 2015 | 51-200 | ✅ |  |  |
| **Deledao** | Santa Clara, California | 2017 | 11-50 | ✅ |  |  |
| **Gaggle** | Dallas, Texas | 1999 | 51-200 | ✅ |  |  |
| **GoGuardian** | El Segundo, California | 2014 | 501 - 1000 | ✅ |  |  |
| **Lightspeed Systems** | Austin, Texas | 1999 | 201-500 | ✅ |  |  |
| **Linewize by Qoria** | San Diego, California | 2015 | 51-200 | ✅ | ✅ |  |
| **Managed Methods** | Boulder, Colorado | 2013 | 11-50 | ✅ |  |  |
| [**Navigate360**](https://navigate360.com/solutions/detect/) | Richfield, Ohio | 2020 | 201-500 | ✅ |  |  |
| **Netsweeper** | Waterloo, Ontario | 1999 | 51-200 | ✅ |  | ✅ |
| **Safer Schools Together** | Surrey, British Columbia | 2008 | 11-50 | ✅ |  |  |
| [**Securly**](https://www.securly.com/) | San Jose, California | 2012 | 201-500 | ✅ | ✅ |  |
| **Sergeant Laboratories** | Onalaska, Wisconsin | 1997 | 51-200 | ✅ |  |  |
