# Supplementary material for: School-Based Online Surveillance of Youth: Systematic Search and Content Analysis of Surveillance Company Websites
Source: J Med Internet Res. 2025 Jul 8;27:e71998. doi: 10.2196/71998 (PMC12262101; doi:10.2196/71998)
Supplement: Multimedia Appendix 3 [file jmir-v27-e71998-s003.docx]

| Company | **Content Features** | **Alert Features** | | **Tracking Features** | | |
| --- | --- | --- | --- | --- | --- | --- |
|  | Anti-Harassment Content Nudge | Company Staff On Call After Hours | After Hours Alerts for School Admins | Alert Management Dashboard | Student Wellness/Risk Scores | Parent Platform |
| Ativion |  | ✅ | ✅ | ✅ | ✅ |  |
| Bark |  | ✅ | ✅ | ✅ | ✅ | ✅ |
| Blocksi |  | ✅ | ✅ | ✅ |  | ✅ |
| Deledao |  |  |  | ✅ |  | ✅ |
| [Gaggle](https://www.gaggle.net/safety-management) |  | ✅ | ✅ | ✅ |  |  |
| [GoGuardian](https://www.goguardian.com/safety-security) |  | ✅ | ✅ | ✅ |  |  |
| [Lightspeed Systems](https://www.lightspeedsystems.com/?utm_source=google&utm_medium=paid-ad&utm_campaign=7013n000001Q7j7&utm_content=brand&utm_term=lightspeed%20systems&gclid=EAIaIQobChMI0K-LyeqlgQMVVB19Ch0Dew3MEAAYASAAEgJLxfD_BwE) |  | ✅ | ✅ | ✅ |  | ✅ |
| [Linewize](https://www.linewize.com/) by Qoria |  | ✅ | ✅ | ✅ | ✅ | ✅ |
| Managed Methods |  |  | ✅ | ✅ |  |  |
| [Navigate360](https://navigate360.com/solutions/detect/) |  |  |  | ✅ |  |  |
| Netsweeper |  | ✅ | ✅ | ✅ |  |  |
| Safer Schools Together |  | ✅ |  |  |  |  |
| [Securly](https://www.securly.com/) | ✅ | ✅ | ✅ | ✅ | ✅ | ✅ |
| Sergeant Laboratories^a^ |  |  |  |  |  |  |
| **Total** | **1** | **10** | **10** | **12** | **4** | **6** |

^a^ Sergeant Laboratories had incomplete information on their website. We reached out to the company for more information, and they ultimately declined to comment.
